# Riverine Bacterial Communities Reveal Environmental Disturbance Signatures within the *Betaproteobacteria* and *Verrucomicrobia*

**DOI:** 10.3389/fmicb.2016.01441

**Published:** 2016-09-15

**Authors:** John Paul Balmonte, Carol Arnosti, Sarah Underwood, Brent A. McKee, Andreas Teske

**Affiliations:** Department of Marine Sciences, The University of North Carolina at Chapel HillChapel Hill, NC, USA

**Keywords:** bacterial community, river, *Betaproteobacteria*, *Verrucomicrobia*, 16S rRNA gene

## Abstract

Riverine bacterial communities play an essential role in the biogeochemical coupling of terrestrial and marine environments, transforming elements and organic matter in their journey from land to sea. However, precisely due to the fact that rivers receive significant terrestrial input, the distinction between resident freshwater taxa vs. land-derived microbes can often become ambiguous. Furthermore, ecosystem perturbations could introduce allochthonous microbial groups and reshape riverine bacterial communities. Using full- and partial-length 16S ribosomal RNA gene sequences, we analyzed the composition of bacterial communities in the Tar River of North Carolina from November 2010 to November 2011, during which a natural perturbation occurred: the inundation of the lower reaches of an otherwise drought-stricken river associated with Hurricane Irene, which passed over eastern North Carolina in late August 2011. This event provided the opportunity to examine the microbiological, hydrological, and geochemical impacts of a disturbance, defined here as the large freshwater influx into the Tar River, superimposed on seasonal changes or other ecosystem variability independent of the hurricane. Our findings demonstrate that downstream communities are more taxonomically diverse and temporally variable than their upstream counterparts. More importantly, pre- vs. post-disturbance taxonomic comparison of the freshwater-dominant *Betaproteobacteria* class and the phylum *Verrucomicrobia* reveal a disturbance signature of previously undetected taxa of diverse origins. We use known traits of closely-related taxa to interpret the ecological function of disturbance-associated bacteria, and hypothesize that carbon cycling was enhanced post-disturbance in the Tar River, likely due to the flux of organic carbon into the system associated with the large freshwater pulse. Our analyses demonstrate the importance of geochemical and hydrological alterations in structuring bacterial communities, and illustrate the response of temperate riverine bacteria on fine taxonomic scales to a disturbance.

## Introduction

Microbial communities form the basis of food webs and regulate biogeochemical cycles on both local and global scales; their function depends on the combined metabolic potential of their members, their gene expression patterns, and their responses to environmental conditions (Fuhrman, 2009). Numerous studies have focused on characterizing natural microbial assemblages to understand which metabolic and biogeochemical processes are likely at play in an ecosystem (McCarren et al., 2010; Martinez-Garcia et al., 2012), as well as to investigate the manner in which environmental conditions structure microbial communities (Fuhrman et al., 2006; Kent et al., 2007). Microbes, particularly in freshwater and marine ecosystems, exhibit biogeographical structure and seasonality due to natural variations in environmental conditions (Crump and Hobbie, 2005; Fuhrman et al., 2006). Changes in environmental conditions due to disturbances—uncommon or irregular events unrelated to, but may co-occur with, seasonal variations—could therefore induce alterations in microbial community composition. Pulse and press disturbances, which cause sudden and gradual changes in natural conditions, respectively, could affect microbial communities in different ways (Allison and Martiny, 2008; Shade et al., 2012).

The effects of disturbance on microbial communities have been investigated in terrestrial (Allison and Martiny, 2008; Shade et al., 2012), and freshwater ecosystems (Jones et al., 2008, 2013; Shade et al., 2012). These studies suggest that microbial communities are generally sensitive to natural or manipulated perturbations, and are quick to respond and change in composition—sometimes in predictable ways (Jones et al., 2008). Consequently, the loss, emergence, or proliferation of different microbial taxa may result in a shift in the community's function. Resilient microbial communities are sensitive to perturbations, but return swiftly to their pre-disturbance state. Alternatively, a resistant microbial community retains its composition regardless of changing conditions, due to the physiological flexibility and adaptability of the microbial taxa that comprise the community (Allison and Martiny, 2008; Shade et al., 2012).

Comparatively few studies have focused on microbial community response to perturbations in rivers. Observational investigations—which focus on natural disturbances—are even less common (Sinigalliano et al., 2007; Amaral-Zettler et al., 2008), as most studies of disturbance are manipulated (Allison and Martiny, 2008; Shade et al., 2011, 2012). As a result, knowledge about the manner in which riverine bacterial communities respond to natural perturbations is limited. Studies conducted following Hurricanes Rita and Katrina demonstrated a shift in microbial pathogens (Sinigalliano et al., 2007) and microbial communities attributed to inputs of sediments, soils, and sewage into waterways (Amaral-Zettler et al., 2008); the focus of these studies, however, were not in rivers. In a saline cyanobacterial mat, initially rare taxa were found to dominate post-hurricane, coinciding with a community functional shift (Yannarell et al., 2007). These studies point to the paucity of information on disturbance responses specifically in riverine bacterial communities compared to other aquatic systems. Yet, understanding community response in rivers is particularly important due to the key roles rivers play in transforming and transporting sediments, nutrients, and organic matter across landscape gradients and from land to the ocean (Battin et al., 2008). As shifts in microbial communities may have consequences for ecosystem health (Mao-Jones et al., 2010) and cycling of nutrients and elements (Battin et al., 2008), the effects of disturbance on community composition must be better understood.

**Figure 1:**
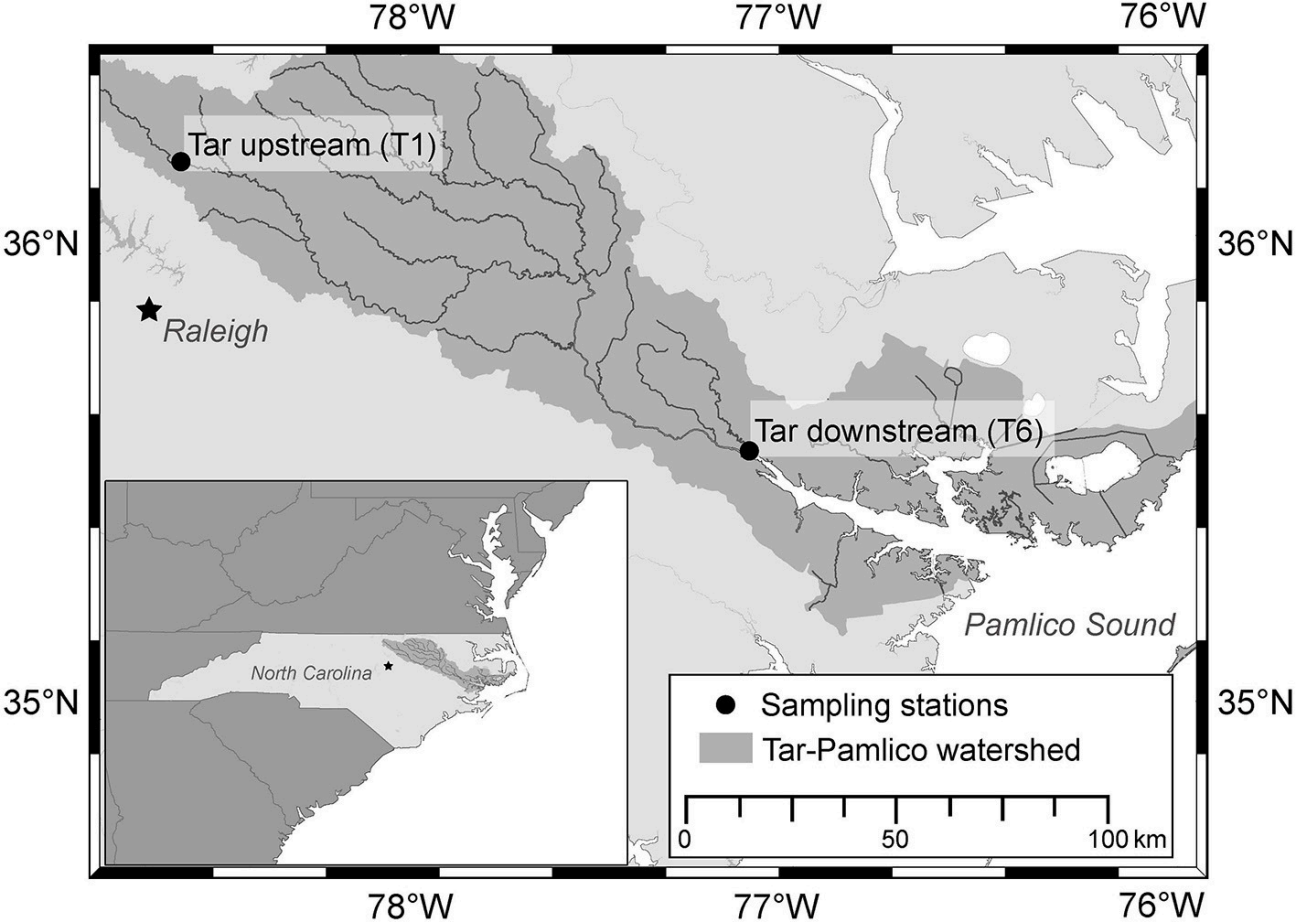
**Tar River Watershed showing the upstream and downstream sampling sites, T1 and T6, respectively**. The inset shows the location of the Tar-Pamlico watershed within North Carolina.

In this study, we assessed the composition of bacterial communities over a 1-year period in the Tar River, a river in eastern North Carolina that drains into Pamlico Sound, the second largest estuarine system in the United States (Paerl et al., 2002). The study was part of a larger project aimed to understand the temporal and spatial dynamics of—and environmental factors that shape—bacterial community composition and enzymatic activities in temperate rivers (Bullock, 2014). At six times over the course of a year, we sampled two stations, upstream near the head of the Tar River and downstream where the river feeds into the estuary (Figure 1). Our sampling included a post-Hurricane Irene collection at the downstream station, shortly after the hurricane inundated the lower portions of the Tar River on August 27, 2011. We posit that the hurricane—which induced hydrological and geochemical alterations—represents a natural environmental disturbance superimposed on seasonal changes in the river. The long-term record of downstream Tar River indicates that the highest water level measured—since the beginning of USGS measurements at this site—corresponded with the landfall of Hurricane Irene, above flood stage (nwis.waterdata.usgs.gov). Since flooding events increase floodplain-river connectivity (Junk et al., 1989), floodplains are likely sources of water, sediment, and new microbial populations to the Tar River following Hurricane Irene. In addition, bacteria from anthropogenic runoff and wastewater can be expected (Sinigalliano et al., 2007; Amaral-Zettler et al., 2008).

With the goal to precisely identify specific bacterial taxa and establish a reference 16S rRNA gene dataset for the Tar River, as well as to tease apart freshwater lineages from putatively allochthonous taxa referencing the well-curated freshwater bacterial database (Newton et al., 2011), we sequenced 16S rRNA genes for fine-scale taxonomic analyses. Since the sampling date range was limited to 1 year (November 2010 to November 2011), there was no baseline comparison for a complete non-disturbed yearly cycle; our study focuses therefore on the time horizon of the pulse effects before and immediately after the disturbance. We focus on the taxonomy and known ecological traits of closely-related taxa within the class *Betaproteobacteria* and phylum *Verrucomicrobia* to demonstrate how disturbance-associated alteration are evident on fine taxonomic scales.

## Materials and methods

### Sampling sites and dates

The Tar River runs for 180 river miles from Triple Springs, NC (36.42°N, 78.80°W) to Washington, NC (35.55°N, 77.08°W), where the water drains into the Pamlico Sound (Powell, 1968). It is characterized by a rural and agriculturally-dominated watershed, with a population size of 500,000 (North Carolina Division of Water Quality, 2010). Two sites within the Tar River, located upstream (station T1; Oxford, NC, 36.19°N, 78.58°W) and downstream (station T6; Washington, NC, 35.56°N, 77.09°W) were chosen to assess the relationship of microbial communities with distinct riverine flow regimes, sediment load, and organic carbon input in the upper Piedmont and in the coastal plain (Figure 1; Bullock, 2014). Due to the close proximity of our sampling sites to USGS survey sites, gauge height, and discharge measurements are available (nwis.waterdata.usgs.gov). Surface water samples were collected from both sites during five sampling events to examine the seasonal dynamics of microbial communities: November 18, 2010 (T6) and November 30, 2010 (T1); February 14, 2011 (T6) and February 16, 2011 (T1); June 20, 2011 (T1) and June 22, 2011 (T6); September 12, 2011 (T1) and September 14, 2011 (T6); November 14, 2011 (T1) and November 15, 2011 (T6). An additional sample was collected at Stn. T6 on August 29, 2011, 2 days after Hurricane Irene crossed the eastern region of North Carolina.

### Sample collection and filtration

At each sampling event, pre-rinsed cubitainers were used to collect approximately 5 L of surface water, of which 500 mL was filtered within 24 h of collection using a vacuum pump through two 45 mm diameter 0.2 μm pore size Nucleopore filters (Track-Etch Membrane Whatman filter). The filters were kept in sterile 15 mL centrifuge tubes at −80°C until extraction of DNA.

### DNA extraction

Total nucleic acids were extracted based on the cetyltrimethylammonium (CTAB) protocol (Dempster et al., 1999) with the following modifications. Sample filters were placed in 2 mL centrifuge tubes (one filter per tube) containing 1 mL of CTAB and frozen overnight at −20°C. Frozen tube samples were thawed at room temperature, and 8 μL of 0.4% (vol/vol) 2-mercaptoethanol was added per tube. The tubes were incubated at 65°C for 15 min with occasional tube inversion. Once the tubes were cooled to room temperature, an equal volume of 24:1 chloroform:isoamyl alcohol was added to each tube. Using a rotating platform, the tubes were shaken for 20 min at room temperature. To separate the aqueous layer from the solution, the tubes were centrifuged at 12,500 rpm at 4°C. The aqueous layer was transferred to a new 2 mL centrifuge tube, and was subjected to one more extraction using an equal volume of chloroform:isoamyl alcohol. After another centrifugation step at 12,500 rpm and 4°C, the aqueous layer was split and placed in two new 1.5 mL centrifuge tubes, to which a half volume of 5 M NaCl and an equal volume (to aqueous layer) of isopropanol were added. After inversion, the tubes containing the treated aqueous layer were incubated at −80°C for 1–2 h. To precipitate the DNA out of the solution, the tubes were centrifuged at 12,500 rpm for 45 min at 4°C. The resulting pellet was washed using 500 μL 70% ethanol, centrifuged for 5 min at 12,500 rpm, air-dried, then resuspended until full dissolution in 50 μL RNAse-free water. The DNA solution was stored in −80°C until further use.

### PCR amplification, cloning, and sequencing of 16S rRNA genes

PCR amplification of the bacterial 16S rRNA gene was performed using a Bio-Rad iCycler thermal cycler (Bio-Rad, Hercules, CA). The PCR mixture contained 2.5 μL of 10X Fast Buffer 1 (TaKaRa, Clontech Laboratories, Inc., Mountain View, CA, USA), 2.0 μL deoxynucleotide triphosphate (dNTP) mix containing 2.5 mM of dNTP (TaKaRa), 2.0 μL each for bacterial primers 8f and 1492r (Teske et al., 2002), 1.0 μL bovine serum albumin (10 mg ml^−1^), 0.25 μL SpeedSTAR *Taq* polymerase (TaKaRa), 1.0 μL DNA template, and 14.25 μL RNA-free water. The PCR conditions used are as follows: initial denaturation round at 94°C, followed by 25 cycles containing a denaturation step at 98°C for 10 s, an annealing step at 60°C for 15 s, and an extension step at 72°C for 10 min, and a final extension round at 72°C for 10 min. Aliquots of the PCR products, including positive and negative controls, were SYBR green-stained and visualized using a 1.5% agarose gel. After confirming the correct amplicon size and sufficient yield, the PCR products were purified using the MinElute® PCR Purification Kit (Qiagen, Valencia, CA) following the MinElute PCR Purification Microcentrifuge and Vacuum Protocol.

The PCR products were cloned into OneShot® TOP10 competent Cells (Invitrogen, Carlsbad, CA) using the TOPO TA Cloning® Kit for Sequencing (Invitrogen) using standard protocol, but modified so that the cloning reaction is incubated for 2 h at room temperature. Transformed cells were grown on LB/Xgal/Kanamycin plates, and the resulting white bacterial colonies were picked and re-plated for sequencing (GENEWIZ, Inc., South Plainfield, NJ) using vector-based M13F/R primers.

### Sequence analyses and accession numbers

Nearly full-length 16S rRNA gene sequences for all of the sampling periods except for the June 2011 upstream and downstream clone libraries were retrieved from GENEWIZ and were assembled into contigs using Sequencher (Gene Codes Corp). The June 2011 clone libraries contained partial-length sequences of approximately 700 bp. The software tools SINA (v1.2.9) was used to initially align the assembled contigs, and Bellerophon version 3 available online (Huber et al., 2004) through the curated 16S rRNA gene database Greengenes (DeSantis et al., 2006) was used to check for chimeras among the contigs. Chimera sequences were removed, and high-quality sequences were uploaded on SILVA online for automated initial alignment of the sequences (Pruesse et al., 2007). The resulting sequences were then uploaded into the ARB software version 95 (Ludwig et al., 2004). All sequences have been submitted to GenBank under accession numbers KP686568 to KP687232.

### Inference of phylogenetic trees

Whenever possible, the closest cultured relatives of uncultured Tar River phylotypes were identified in the SILVA reference database and were included to create phylogenetic trees; otherwise, the most closely-related environmental clone sequences were included in the tree. Many of the sequences used in the final phylogenetic trees were retrieved from the Freshwater Lake Bacteria ARB Database (Newton et al., 2011), and from previous analyses of the freshwater genera *Limnohabitans* (Kasalický et al., 2013) and *Polynucleobacter* (Wu and Hahn, 2006). Final alignments were edited manually, using *Escherichia coli* secondary structure as filter (Pruesse et al., 2007). The final phylogenetic trees were inferred using the Maximum Likelihood algorithm and the general time-reversible gamma distributed rate variation model (GTRGAMMA) as implemented in ARB; bootstrap values were calculated using ARB's Rapid Bootstrap Analysis tool. Consensus trees with collapsed polytomies were based on 1000 bootstrap runs. As the June 2011 sequences were only partial-length, they were added manually to the resulting trees using the Quick Add Marked feature of ARB Parsimony; filters were applied depending on the length of each partial-length sequence. Branching nodes with corresponding bootstrap support of ≥60% were considered validated (Peplies et al., 2008); bootstrap values for poorly-supported nodes are not shown.

### OTU analyses using MOTHUR

Sequences were also imported into MOTHUR (v.1.28.0), aligned using the SILVA reference database within the package, and were clustered based on 97 or 95% sequence similarity for further OTU-based analyses (Schloss et al., 2009). Because the sequences imported into MOTHUR had been manually curated on ARB, no sequences were excluded from the OTU-based clustering step on MOTHUR.

### Multivariate analysis of community dissimilarity and environmental parameters

Data produced from MOTHUR were used to analyze community dissimilarity using the *Vegan* package on R (version 2.3.0, http://r-forge.r-project.org/projects/vegan/). Dissimilarity matrices were calculated using the Bray-Curtis and Morisita-Horn indices. The Morisita-Horn index was chosen to complement the more commonly used Bray-Curtis index to address the relatively uneven sequencing depths of the clone libraries. The presence-absence-based Sørensen index was also used for initial analysis but was not included due to the poor discriminatory power of the results. We used an OTU cutoff of 95% sequence similarity to avoid the high frequency of zero values associated with setting the cutoff at 97%. The dissimilarity matrices were visualized using non-metric multidimensional scaling (NMDS). The function *adonis* in the *Vegan* package was used to run Permutational MANOVA (PerMANOVA) with 999 permutations and explore whether variations in community composition can be explained by environmental factors. We included the following factors in the PerMANOVA analysis: sampling site, hurricane status, salinity, dissolved oxygen (DO) and pH.

## Results

### Environmental conditions

**Figure 2:**
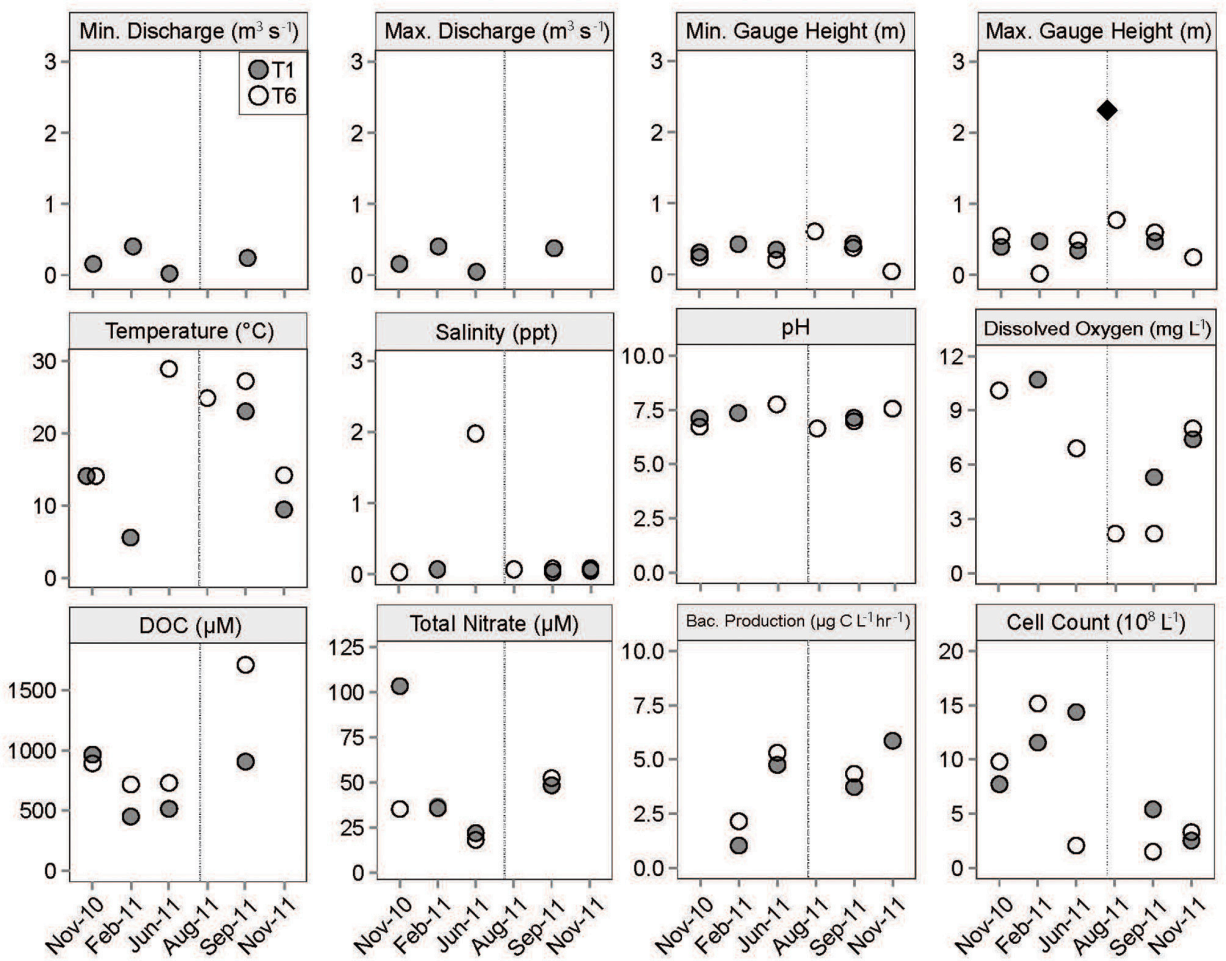
**Physical, chemical, and biological parameters from the Tar River at Stn. T1 (upstream) and Stn. T6 (downstream)**. The diamond in the maximum gauge height plot represents the water level (2.30 m, source: USGS) immediately after the landfall of Hurricane Irene (indicated by the vertical dashed line) on August 27, 2011. DOC, cell counts, and bacterial production data are from a separate study (Bullock, 2014).

Throughout the entire sampling period, the Tar River experienced drought conditions compared to a 20-year precipitation average in the basin (USGS, waterdata.usgs.gov/nwis). Rainfall within 3 days of sample collection occurred only at three sampling dates—November 2010, August 2011, and September 2011 (Table S1). Salinity values indicated fresh conditions throughout the study for both sites, with the exception of a salinity peak downstream in June 2011 (Figure 2), indicating brackish conditions. Discharge was highest in February 2011 and September 2011 upstream. Discharge data are not available downstream at Stn. T6, but these data are available from a station immediately upstream (T5) where a range of physical and chemical parameters as well as microbial activities were measured (Bullock, 2014). Water level (measured through gauge height) was highest upstream in February 2011; the high level measured downstream in late August 2011, above flood stage, was the result of heavy precipitation associated with Hurricane Irene. Bacterial production and cell counts showed large temporal variability (Figure 2), as discussed in greater detail in another study (Bullock, 2014).

### Year-round bacterial community composition upstream and downstream

**Figure 3:**
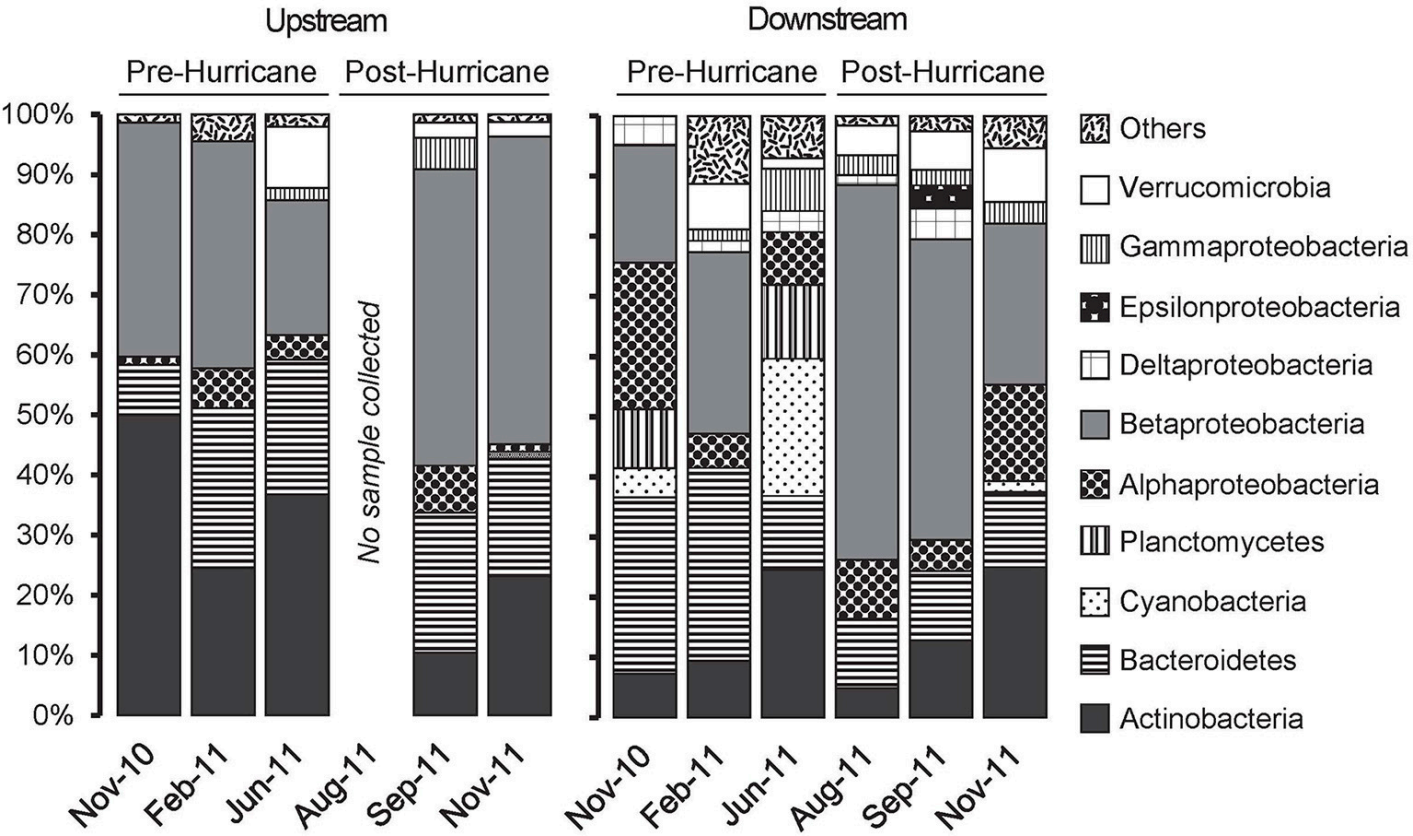
**Tar River clone libraries from November 2010 to November 2011**. *Actinobacteria* is in dark gray, *Betaproteobacteria* is in light gray, and *Verrucomicrobia* is in white. “Others” include *Armatimonadetes, Chlorobium, Chloroflexi, Spirochaetae* as well as candidate phyla OD1, OP3, OP11, TM6, and TM7.

After quality filtration and removal of chimeras, a total of 564 nearly full-length and 102 partial-length (800–900 bp) 16S rRNA gene sequences were retrieved from eleven 16S rRNA gene clone libraries, and used for microbial community investigations and downstream statistical analyses. The most abundant phylotypes belonged to the *Actinobacteria, Bacteroidetes, Alphaproteobacteria* and *Betaproteobacteria*, respectively though their relative contribution varied throughout the study (Figure 3). *Betaproteobacteria* increased in relative clone library contribution after the disturbance. Major bacterial groups—*Bacteroidetes, Alphaproteobacteria, Deltaproteobacteria*, and *Gammaproteobacteria*—retained a similar downstream clone library distribution before and after the disturbance. The *Actinobacteria* decreased in relative clone library contribution from upstream to downstream in November 2010 and February 2011; only in late August 2011 and persisting into the last sampling date did the actinobacterial contribution to the clone libraries appear roughly equal upstream and downstream (Figure 3). Members of the *Gammaproteobacteria* and *Cyanobacteria* were detected predominantly downstream, while *Planctomycetes* were found exclusively downstream (Figure 3). Members of the *Deltaproteobacteria, Epsilonproteobacteria, Verrucomicrobia*, and other phylum-level lineages (grouped together as “Others,” including members of *Armatimonadetes, Chloroflexi, Chlorobium, Spirochaetes* and of candidate divisions OD1, OP3, OP11, TM6, and TM7) appeared only sporadically and comprised a small fraction of the total sequences (Figure 3). As a caveat, these clone library contributions offer at best a relative approximation of bacterial abundances of the most dominant phylotypes, and would require explicitly quantitative controls, such as qPCR quantifications or FISH counts, for validation.

**Figure 4:**
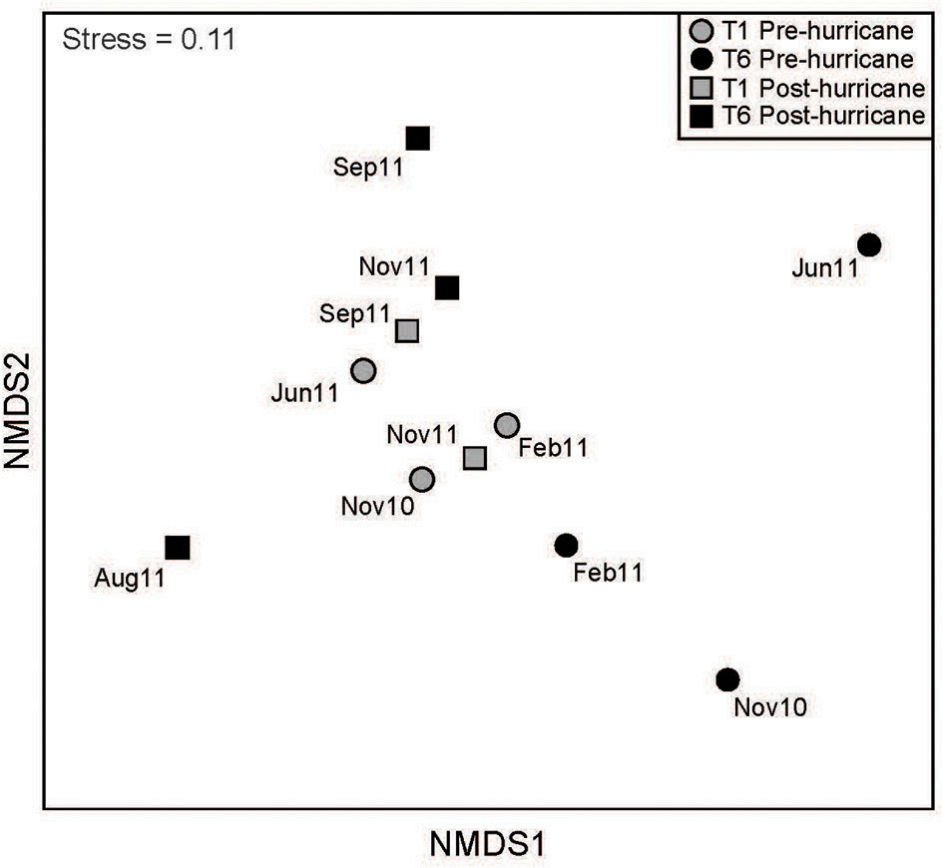
**Non-metric Multidimensional Scaling (NMDS)**. Bray-Curtis dissimilarity index was used to calculate the dissimilarity matrix for bacterial community composition at an OTU cutoff of 0.05.

A wider range of bacterial phyla and phylotypes was detected downstream than upstream (Figure 3), with very few shared OTUs at the 97% sequence similarity cutoff (Figure S1). Analyses with MOTHUR revealed a greater number of OTUs downstream throughout the entire sampling period (Figure S1). NMDS using Bray-Curtis and Morisita-Horn dissimilarity indices on OTUs (cutoff = 0.05) revealed that upstream communities clustered tightly, whereas the high scatter in downstream communities indicated high community dissimilarity across the sampling periods (Figure 4). PerMANOVA of the clone libraries using Bray Curtis and Morisita-Horn indices indicate that sampling site is significantly correlated with variations in community composition (Bray-Curtis: *R*^2^ = 0.18, *p* = 0.013; Morisita-Horn: *R*^2^ = 0.18, *p* = 0.007; Table S2).

### Post-disturbance differences in bacterial community composition

While bacterial community composition upstream remained similar throughout the sampling period, the downstream clone library from the August 2011 sample (collected 2 days after Hurricane Irene) showed a bacterial community structure distinct from other downstream clone libraries (Figure 3). Representation of the *Proteobacteria*, in particular the *Betaproteobacteria*, increased in the downstream clone libraries from June 2011 to August 2011, 2 days post-hurricane; in contrast, the *Actinobacteria* decreased in relative proportion (Figure 3). In addition, the *Cyanobacteria* were undetected immediately post-disturbance and reappeared in November 2011 in small clone library proportions (Figure 3). Furthermore, PerMANOVA indicates that disturbance status—before vs. after—(Bray-Curtis: *R*^2^ = 0.23, *p* = 0.003; Morisita-Horn: *R*^2^ = 0.39, *p* = 0.004; Table S2) and salinity (Bray-Curtis: *R*^2^ = 0.18, *p* = 0.037; Morisita-Horn: *R*^2^ = 0.26, *p* = 0.026; Table S2) significantly correlate with community composition.

For a fine-scale taxonomic analysis of environmental disturbance after Hurricane Irene on the bacterial communities, we selected the well-characterized *Betaproteobacteria* and the *Verrucomicrobia*. We identified individual families, genera and species of cultured bacteria, or well-defined clusters of phylotypes, and used their known ecological niches in context with hydrological and geochemical data to develop hypotheses for their changing detection patterns. Ecophysiological inferences were based on the presence/absence or relative increase/decrease of clones closely related to taxa within the *Betaproteobacteria* and *Verrucomicrobia*, with the added caveat that even bacteria with identical or near-identical 16S rRNA gene sequences can have differing genomes and physiologies (Jaspers and Overmann, 2004).

### Families of the *Betaproteobacteria*

**Figure 5:**
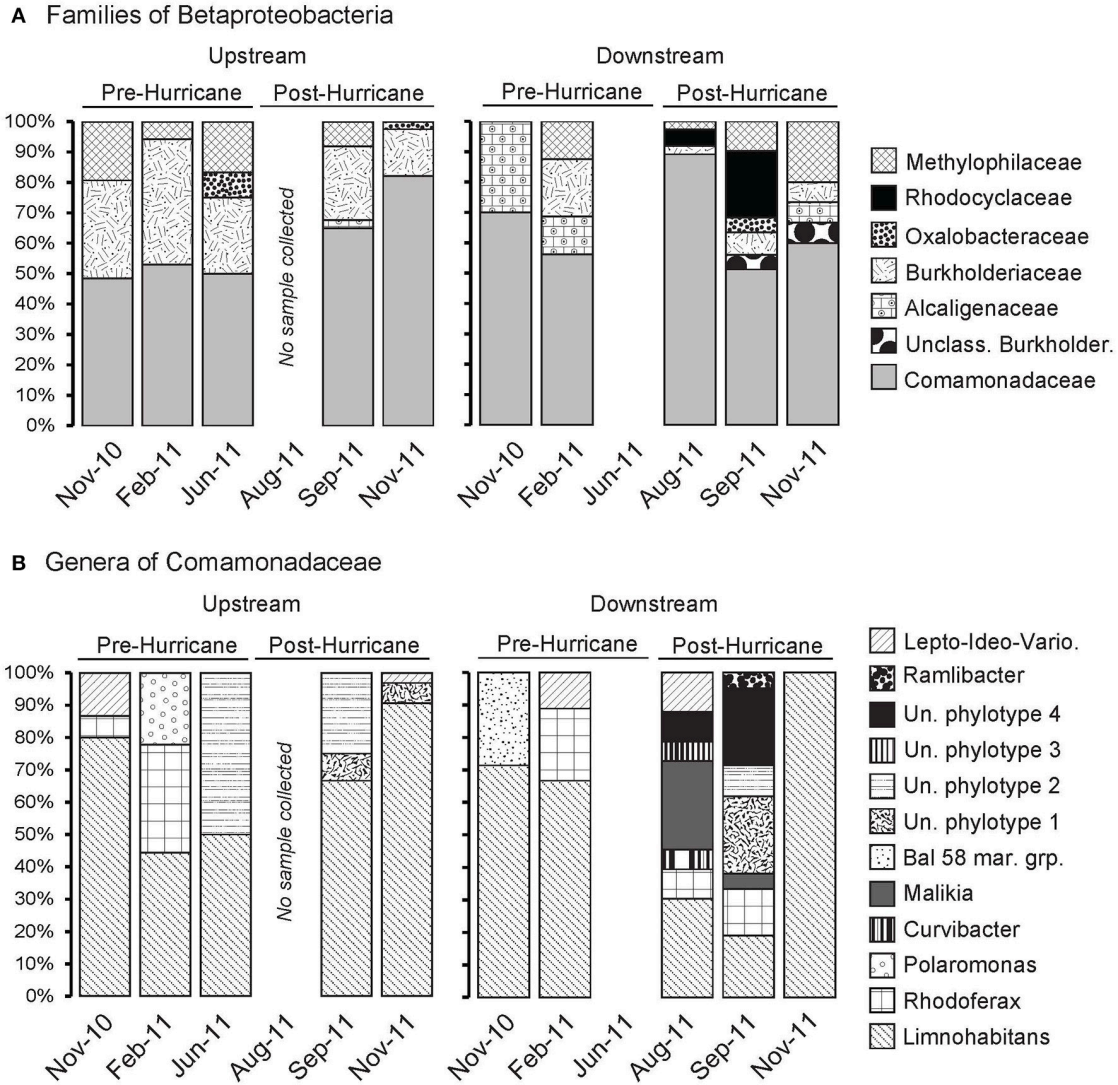
**Relative contributions of families within the *Betaproteobacteria* (A) as well as genera and unassigned phylotypes within *Comamonadaceae* (B)**. The identification of the different families, genera and well-defined clusters in *Betaproteobacteria* was based on constructed phylogenetic trees shown in Figure S2A (families) and Figure S2B (genera and other clusters), their annotation following published taxonomies of betaproteobacterial families and genera. “Un. Phylotype” refers to the Unassigned phylogtypes found within *Comamonadaceae*.

*Betaproteobacteria* constituted the largest fraction of detected proteobacterial clones, with the exception of the June downstream 2011 clone library, where *Betaproteobacteria* clones were not detected (Figures 3, 5A,B). Six betaproteobacterial families—five of which contain the “bet” lineages in the Freshwater Lake Bacteria database (Newton et al., 2011)—were identified in the downstream sequence data sets: *Comamonadaceae* (betI), *Burkholdericeae* (betII), *Alcaligenaceae* (betIII), *Methylophilaceae* (betIV), *Oxalobacteraceae* (betVII), and *Rhodocyclaceae*. Of these, only *Rhodocyclaceae*—detected strictly downstream, post-disturbance—had no lineages included in the freshwater bacterial database (Newton et al., 2011). Only four of these families—*Comamonadaceae, Burkholderiaceae, Methylophilaceae*, and *Oxalobacteraceae*—were consistently detected upstream (Figures 5A,B; Figures S2A,B). The cosmopolitan freshwater genus *Limnohabitans* (Hahn et al., 2010) in the family *Comamonadaceae* constituted the largest fraction (38%) of detected betaproteobacterial sequences in the clone libraries, appearing in all but the downstream June 2011 clone library (Figures 5A,B). Within the genus *Limnohabitans* (Figure S3), the Tar River clones were mostly members of previously established, phylogenetically defined subgroups termed tribes (Newton et al., 2011) or lineages (Kasalický et al., 2013). Other genera and lineages in the family *Comamonadaceae* were not widely detected in the Tar River, and some were found only in samples collected after Hurricane Irene. Clones related to the genus *Polynucleobacter* (Hahn, 2003)—belonging to the betII lineage (Newton et al., 2011) within the family *Burkholderiaceae*—comprised the second most abundant (17%) betaproteobacterial group in the clone libraries, with representative clones in all but two (November 2010 and June 2011) downstream clone libraries (Figure S4).

### Post-disturbance changes within the *Betaproteobacteria*

The post-disturbance, downstream betaproteobacterial clones, particularly those collected after the passage of Hurricane Irene in late August 2011 and September 2011, differed in family and genus level-composition from those in pre-disturbance clone libraries (Figures 5A,B, Figures S2A,B). Specifically, betaproteobacterial groups that were not detected in pre-disturbance clone libraries prior to the hurricane were observed post-disturbance, including members of the *Rhodocyclaceae* family, an unclassified *Burkholderiales* group, as well as several genera and unassigned phylotypes within the ubiquitous *Comamonadaceae*. Clones identified as belonging to *Rhodocyclaceae* most closely related to the obligately or facultatively anaerobic genus *Ferribacterium* (Cummings et al., 1999) were only detected downstream and post-disturbance. Clones of the *Methylophilaceae*—a family of aerobic methylotrophic bacteria (Garrity et al., 2005)—closely associated with the newly designated *Candidatus* Methylopumilus planktonicus (previously LD28) were only detected downstream post-disturbance, from late August 2011 to November 2011 (Figure S5), whereas related *Methylophilaceae* clones related to *Candidatus* Methylopumilus turicensis (previously PRD01a001B) were found throughout the sampling period upstream and downstream (Salcher et al., 2015).

Additional diversity within the *Comamonadaceae* was detected downstream and post-disturbance (Figure 5B). The genus *Malikia*, which consists of isolates primarily from activated sludge (Spring et al., 2005), appeared in the late August and September 2011 downstream clone libraries. A significant fraction of the August 2011 clones within *Comamonadaceae* remained unclassified. Phylogenetic analysis using ARB revealed that these clones lacked bootstrap support for placement into specific phylogenetic branches of the *Comamonadaceae*; therefore we refer to these clones as Unassigned phylotype assemblages 1, 2, 3, and 4 (Figure 5B; Figure S2B). In the downstream clone libraries, these unassigned phylotypes were found consistently in post-hurricane samples. Members of Unassigned phylotype assemblages 1 and 2 also appeared in upstream samples; only members of Unassigned phylotype assemblages 3 and 4 emerged strictly downstream post-disturbance. While clones of Unassigned phylotype assemblage 3 only appeared in August 2011, those in Unassigned phylotype assemblage 4 persisted into September 2011 (Figure 5B; Figure S2B).

### Lineages within *Limnohabitans* and *Polynucleobacter*

We examined whether a lineage-specific disturbance response was present within the two most abundant betaproteobacterial genera in the clone libraries. The genus *Limnohabitans* was originally subdivided into phylogenetically defined subclusters, termed tribes (Newton et al., 2011). After a substantial cultivation effort, this scheme was revised and the entire genus, including new cultures, was reclassified into different lineages Lim A, LimB, LimC, LimD, and LimE (Kasalický et al., 2013). The *Limnohabitans*-related sequences in our dataset are affiliated with the lineages LimA, LimB, LimC, and LimD. The Tar River clones are widespread among the *Limnohabitans* lineages, but only the LimB and LimD lineages contained strictly post-disturbance clones from upstream and downstream sampling sites (Figure S3).

In contrast, downstream post-disturbance clones within *Polynucleobacter* are confined to a single phylogenetically defined lineage, PnecC—members of which are quick to respond to allochthonous DOC (Hutalle-Schmelzer and Grossart, 2009). However, because all detected downstream *Polynucleobacter* clones occurred only within PnecC (Figure S4), a lineage-specific disturbance signature cannot be presently identified in the genus *Polynucleobacter*. Furthermore, because this group contains substantial genetic and physiological diversity despite having ≥99% similarity in 16S rRNA gene sequences (Hahn et al., 2016), the ecological roles of PnecC-affiliated *Polynucleobacter* populations before and after the disturbance are likely complex.

### Post-disturbance differences within the *Verrucomicrobia*

**Figure 6:**
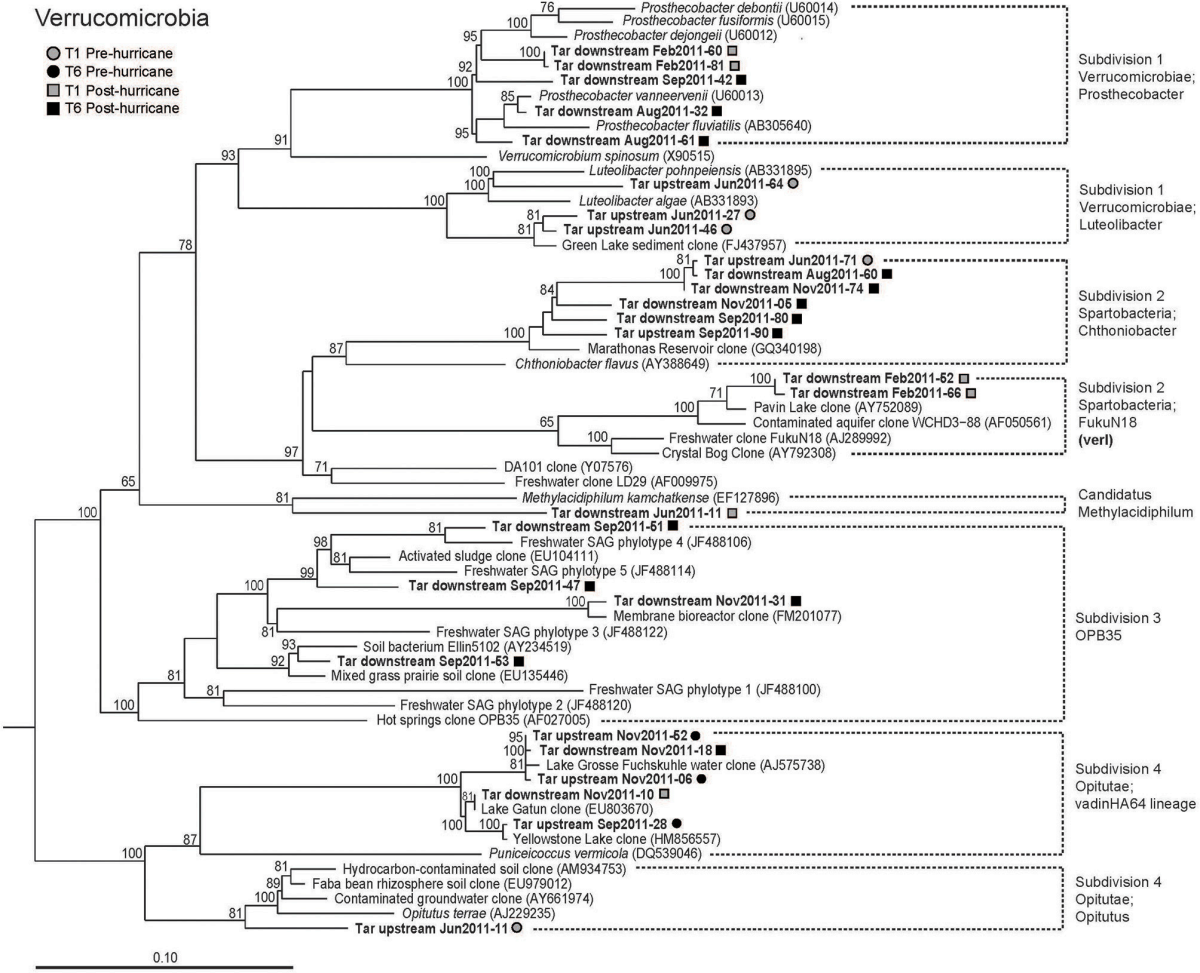
**Phylogeny of subdivisions of the phylum *Verrucomicrobia***. Individual well-defined clusters are identified in the tree. Wherever possible, each identification includes a class and genus name; otherwise, a descriptive name is given to each cluster. “verI” refers to a freshwater lineage within Subdivision 2 as classified by Newton et al. (2011). Clones in bold type font are the Tar River clones; cultured species are italicized. The rapid bootstrap analysis feature of the Maximum Likelihood algorithm was used. The tree is rooted by *Lentisphaera araneosa* (AY3490428) and *Victivallis vadensis* (AY049713).

Although recovered in lesser frequency, the *Verrucomicrobia* maintained their clone library representation but changed in phylogenetic composition post-disturbance both upstream and downstream; they were therefore analyzed as an example of a non-dominant phylum-level lineage recording potential disturbance impact. We were also interested in the *Verrucomicrobia* since their genomes encode the highest number polysaccharide hydrolases in any bacterial phylum, enabling effective substrate degradation in the aquatic environment (Martinez-Garcia et al., 2012). Throughout the entire sampling period, a total of 27 clones were classified as belonging to the phylum *Verrucomicrobia* (Figure 6). These clones consisted of members of four of the seven identified order-level subdivisions within *Verrucomicrobia* (Hugenholtz et al., 1998; Sangwan et al., 2005; Schlesner et al., 2006), including Subdivision 1 (*Verrucomicrobiae)*, Subdivision 2 (*Spartobacteria)*, Subdivision 3 (a lineage including the hot spring clone OPB35; Hugenholtz et al., 1998), and Subdivision 4 (*Opitutacaeae*) as well as the candidate division *Candidatus* Methylacidiphilum (Islam et al., 2008; Figure 6). The FukuN18 freshwater cluster within Subdivision 2 is also referred to as the verI lineage, subdivided into two clades: verI-A and verI-B (Newton et al., 2011).

Pre-hurricane upstream verrucomicrobial clones were related to the genera *Chthoniobacter* in Subdivision 2, *Luteolibacter* in Subdivision 1, or *Opitutus* in Subdivision 4. Pre-hurricane downstream verrucomicrobial clones were only found in February 2011 and belonged to the FukuN18 freshwater group in subdivision 2 (Glöckner et al., 2000; Newton et al., 2006) and the freshwater genus *Prosthecobacter* within subdivision 1 (Hedlund et al., 1997). The post-hurricane verrucomicrobial community represented a different set of lineages: the *Chthoniobacter* group in Subdivision 2, which is commonly dominated by soil bacteria (Sangwan et al., 2005; Freitas et al., 2012), and the vadin HA64 (*Opitutaceae*) lineage of Subdivision 4, consisting of freshwater clones (Burkert et al., 2003; Figure 6). Most interestingly, only downstream, post-hurricane verrucomicrobial clones fell into Subdivision 3, which is represented by a cultured soil isolate, Ellin5102 (Joseph et al., 2003), multiple related soil isolates (Sangwan et al., 2005), as well as freshwater and seawater single cell representatives that were captured during uptake of fluorescently labeled polysaccharides, using fluorescence-activated cell sorting (Martinez-Garcia et al., 2012).

## Discussion

### Upstream and downstream hydrological and community composition differences

The drought-stricken Tar River experienced a lack of hydrological connectivity throughout the study period in 2010 and 2011, evident by the parameters measured upstream and downstream. Briefly, hydrological connectivity refers to water-mediated transfer of matter, energy and/or organisms within and between the hydrologic cycle (Pringle, 2001). We posit that the hydrological disconnect at the two stations contributed to the differences in upstream and downstream bacterial communities, often with minimal overlap of OTUs (Figure S1). Additionally, communities at the upstream station were less temporally variable—indicated by a relatively tight sample clustering (Figure 4)—than their downstream counterparts. The temporally- and spatially-variable bacterial entrainment along the Tar River from its many tributaries also likely shaped the differences in upstream and downstream community composition and dynamics. Furthermore, the greater community diversity downstream suggests the cumulative effect of bacterial input into the Tar River; similar trends of increasing community diversification were observed toward estuarine-influenced sampling sites in the Columbia River (Crump et al., 1999). Accordingly, our statistical analyses show that sampling location accounts for a fraction of the variation in bacterial community composition (Table S2).

The year-round hydrological disconnect and community compositional differences in the sampling stations persisted even after the hurricane. When the salinity at the downstream site peaked at 1.96 ppt in June 2011 (Figure 2), *Betaproteobacteria* were undetected in the clone library; however, in August 2011, this freshwater group became predominant in the clone libraries, coincident with freshening of the downstream waters observed after Hurricane Irene had passed through on August 27, 2011 (Figures 3, 5A,B). Previous studies of the effects of storm events on freshwater microbial assemblages also point to salinity changes (Jones et al., 2013) and precipitation (Tseng et al., 2013) as important structuring factors. The betaproteobacterial community downstream post-hurricane, however, was distinct and harbored greater diversity compared to the upstream community. A similar increase in microbial richness has been observed in a freshwater reservoir following a typhoon (Tseng et al., 2013). These data suggested that members of the *Betaproteobacteria* were thriving in the river and responded to the disturbance in specific ways that should make them rewarding targets for taxon-specific ecological interpretation.

### Ecological interpretation of betaproteobacterial taxonomic profiles

The qualitative and quantitative changes in community structure could likely be explained either by the introduction of allochthonous bacterial groups, a change in environmental conditions conducive to the *in-situ* growth and detection of new groups, or both.

The post-hurricane detection of betaproteobacterial clones within the family *Rhodocyclaceae* closely related to cultured obligately or facultatively anaerobic bacteria, for example, matched a post-disturbance regime of increased carbon loading, hypoxia, and sediment resuspension. Surface water data from downstream in late August 2011 as well as September 2011 indicated hypoxic conditions (2.11 mg/L; Figure 2; Tables S1A,B), and DO correlated negatively with dissolved organic carbon concentrations (data not shown). Such conditions reflect the metabolic demands of heterotrophic microbes following the availability of significant amounts of organic carbon, which promotes oxygen depletion and anoxia in surficial sediments. A downstream August 2011 clone was closely related to *Ferribacterium limneticum*, a strictly anaerobic dissimilatory iron reducer originally isolated from acid-mining impacted lake sediments (Cummings et al., 1999). Several August and September 2011 clones were most closely related to *Dechloromonas agitata* (Figure S2A), a facultative anaerobe capable of dissimilatory reduction of perchlorate (Achenbach et al., 2001), an unusual electron acceptor with a variety of natural sources in the environment (Dasgupta et al., 2005). A clone from the downstream September clone library closely matched the cultured phototroph *Rhodocyclus purpureus* (Pfennig, 1978) which grows in light-exposed, organic-, and nutrient-rich waters (Imhoff, 2006). A similar habitat preference could explain the detection of seven clones of an unclassified *Rhodocyclaceae* lineage (represented by uncultured freshwater clones of diverse geographic origins, and classified as 12up in the SILVA database) in the downstream September clone library (Figure S2A). On the level of the whole bacterial community, analysis with PerMANOVA, however, does not indicate a statistically significant correlation between variations in dissolved oxygen concentration and those in bacterial community composition (Table S2); we interpret this to mean that the bacterial community, as a whole, can tolerate hypoxic conditions, and is not overturned or fundamentally altered by the introduction of specific new community members.

Since closely-related cultured representatives of *Methylophilaceae*—*Candidatus* Methylopumilus planktonicus and *Candidatus* Methylopumilus turicensis—are typically rare in warm waters (Salcher et al., 2015), their sustained detection downstream in the summer months could also be due to changes in carbon source and nutrients in the Tar River. As alpha- and gammaproteobacterial marine methylotrophs have been implicated in heterotrophic DOM degradation following phytoplankton blooms (McCarren et al., 2010), it is possible that the detection of methylotrophs may reflect the die-off stage of summer cyanobacterial blooms that led to the high DOC concentrations measured in September 2011 (Figure 2, Table S1B). However, the large freshwater pulse flushing the Tar River may have obliterated the effects of preceding cyanobacterial blooms or other spatiotemporally localized events, consistent with the observation that no cyanobacterial sequences were found remaining in the clone libraries from late August 2011 or September 2011 samples. Instead, we hypothesize that the *Methylophilaceae* were more likely involved in processing the allochthonous organic matter introduced from tributaries and flood plains into the river.

Members of *Limnohabitans*, the predominant genus of the betaproteobacterial family *Comamonadaceae* in the Tar River dataset, are typical inhabitants of freshwater lakes and rivers (Hahn et al., 2010), although some lineages have been observed in estuaries (Kasalický et al., 2013). We therefore interpret that the detection of diverse *Limnohabitans* lineages in the August 2011 samples resulted from *in-situ* growth following the freshening of the water column near the mouth of the river; the hydrological data downstream show a drop in salinity—from brackish to fresh conditions—from June 2011 to late August 2011 (Figure 2, Table S1B). PerMANOVA shows that changes in salinity account for a fraction of the variations in bacterial community composition (Table S2), in accordance with the major contribution of freshwater betaproteobacterial taxa in and after the freshwater pulse in late August 2011. The specific lineages found downstream only after the disturbance—LimB and LimD–may have responded to selective pressures associated with this event. While LimB contains cultured strains, LimD remains defined only by sequences; clones affiliated with these lineages have a wide environmental distribution (Kasalický et al., 2013). Generally, members of *Limnohabitans* are characterized by swift response to nutrient pulses in mesocosms, resulting in increased growth (Šimek et al., 2006); their abundance is also positively correlated with compounds that serve as proxies for low molecular weight substrates of algal origin (Šimek et al., 2010). Thus, the freshening of the Tar River, combined with organic matter loading, was conducive to the growth of members from various *Limnohabitans* lineages. Furthermore, the detection of diverse *Limnohabitans* lineages even after the disturbance could be a reflection of species- or finer taxonomic-level niche differentiation (Kasalický et al., 2013) that enabled their continued coexistence. Interestingly, *Limnohabitans'* apparent sensitivity to salinity, even at relatively low levels, contrasts with their ecophysiological flexibility to persist in turbid, organic carbon-impacted and hypoxic flood waters, as shown here.

The strictly downstream, post-disturbance detection of the clones related to the genus *Malikia—*with representatives cultured from activated sludge (Spring et al., 2005)—may reflect a joint anthropogenic and environmental disturbance, which introduces allochthonous bacterial species that originate from wastewater treatment plants. The presence of a wastewater treatment plant upriver (Rocky Mount, NC) that could affect the downstream sampling site (T6, Washington, NC) is congruent with our hypothesis. In a potentially analogous marine case, an uncultured betaproteobacterial group closely related to *Malikia* increased following a storm event in the marine waters of Kaneohe Bay, Hawaii (Yeo et al., 2013). The transient presence of clones related to *Malikia* in the August and September clone libraries—undetected in the downstream November 2011 clone library—suggests that this group was either introduced from terrestrial runoff only to be flushed out of the system, or that the gradual shift of the environmental conditions back to pre-disturbance state may not have been conducive to their growth.

### Ecological interpretation of changes in *Verrucomicrobia*

Clone library and high-throughput sequencing surveys indicate that—with few exceptions (Cardman et al., 2014)—*Verrucomicrobia* comprise a minor fraction of aquatic bacterial communities. Yet, the members of this phylum play a potentially significant role in polysaccharide degradation in freshwater and marine habitats. Single-cell genomics of several marine *Verrucomicrobia* cells within Subdivision 1 reveal the highest known abundance—among all bacterial phyla—of genes encoding enzymes involved in carbohydrate degradation, including glycoside hydrolases, sulfatases, carbohydrate esterases, and polysaccharide lyases (Martinez-Garcia et al., 2012). This metabolic flexibility may explain the presence of this verrucomicrobial group among the active polysaccharide degraders in temperate (Martinez-Garcia et al., 2012) as well as in Arctic waters (Cardman et al., 2014). Additionally, the genome of *Chthoniobacter flavus*, the first cultured representative of Subdivision 2, contains many genes required to metabolize a wide array of saccharide components derived from plant biomass (Kant et al., 2011). The consistent detection of *Verrucomicrobia* clones in all post-disturbance samples, upstream and downstream, suggests unusual adaptability that allows for the persistence of *Verrucomicrobi*a.

A phylogenetic analysis of the Tar River clones belonging to *Verrucomicrobia* identified the downstream, post-disturbance clones as clustering with Subdivision 3-affiliated clones; this group contains a terrestrial cultured representative and uncultured members from a wide range of ecosystems (Joseph et al., 2003; Sangwan et al., 2005; McCarren et al., 2010). The hydrologic disconnect upstream and downstream even after the passage of Hurricane Irene suggests that tributaries and runoff from floodplains (Junk et al., 1989) could be potential sources for the Subdivision 3-affiliated clones, which were only identified downstream after the disturbance. Freshwater phylotypes of this group showing high affinity to specific polysaccharide substrates (Martinez-Garcia et al., 2012) belonged to the same phylogenetic cluster as the post-disturbance, downstream *Verrucomicrobia* found in the Tar River (Figure 6). If the Tar River Subdivision 3 clones share the high affinity for polysaccharides that distinguishes their close relatives (Martinez-Garcia et al., 2012), they could have been selected for by the large post-hurricane pulse of dissolved organic matter into the river. The downstream, post-disturbance subdivision 3 clones of the Tar River therefore likely shared a similar ecological niche as members of the *Methylophilaceae*, cycling allochthonous organic carbon. Additionally, the presence of a Subdivision 3 clone (clone Sept2011-53; Figure 6) most closely related to soil clones and distinct from the freshwater Subdivision 3 clones, could be an indicator of terrestrial runoff into the Tar River.

### Indications of return to pre-disturbance state

Although our restricted sampling time window after the hurricane prevents a full assessment of microbial community resilience, the appearance and disappearance patterns of hurricane flood indicators on genus- and family-level taxonomic scales are consistent with the community's return to a pre-disturbance state. For example, the putatively disturbance-associated genus *Malikia* and various unassigned phylotypes—in the betaproteobacterial family of *Comamonadaceae—*were undetectable by November 2011 (Figure 5); these patterns also hold with the obligately and facultatively anaerobic members of *Rhodocyclaceae* (*Ferribacterium, Dechloromonas*, and *Rhodocyclus*) detected downstream, post hurricane, coincident with the return of oxic conditions in the Tar River. These betaproteobacterial community changes gradually decrease the overall representation of *Betaproteobacteria*.

## Conclusion

Our study in the Tar River illustrates taxon-specific responses of riverine microbial communities to a disturbance. The predominance of betaproteobacterial clones and detection of specific verrucomicrobial taxa highlight the ecological importance of specific bacterial groups in light of alterations in riverine physical and geochemical conditions. These findings suggest that Tar River bacterial communities are quick to respond to perturbations, consistent with other studies of microbial response to disturbances (Allison and Martiny, 2008; Jones et al., 2008; Shade et al., 2012). Moreover, we find that the downstream community composition show indications of a return to a pre-disturbance state, in accordance with previous findings that terrestrial and freshwater microbes exhibit resilience following perturbations (Allison and Martiny, 2008; Shade et al., 2012). For example, within *Betaproteobacteria*, many uniquely downstream, post-disturbance clones within the *Comamonadaceae* and the *Rhodocyclaceae* were no longer detected 2 months after the disturbance. Our results align with previous findings that, after pulse disturbances, resilient communities tend to return to the pre-disturbance state; in contrast, press disturbances usually force communities to stabilize at a new mean state (Shade et al., 2012).

Here, we demonstrate that while perturbations impact the whole community, distinct and ecophysiologically informative changes appear on and below the genus level, illustrating the importance of fine-scale taxonomic identification in assessing the impacts of disturbance on microbial population. A close examination of the preferred ecological niche of pure cultures closely related to clones detected in the Tar River strictly post-disturbance illuminated possible roles for these organisms. Specifically, the post-disturbance detection of clones related to betaproteobacterial methylotrophs and verrucomicrobial taxa involved in polysaccharide degradation, in tandem with the observed sharp increase in DOC concentrations after the hurricane, suggested enhanced carbon cycling by specific microbial responders in the lower portions of the Tar River. While the geochemical regime post-disturbance (increased DOC, decreased dissolved oxygen) would be consistent with cyanobacterial bloom die-off, we hypothesize that the large freshwater pulse minimized the impacts of preceding bloom events by flushing the river system—consistent with the disappearance of cyanobacterial sequences from the clone libraries of samples collected 2 days after Hurricane Irene—and that allochthonous carbon input from floodplains and tributaries altered riverine conditions and microbial communities. In addition, identification of wastewater-associated bacteria suggests anthropogenic input into the river, a matter with potentially significant consequences for public health. In future studies of bacterial community temporality and perturbation response, a similar approach of fine-scale taxonomic analysis, coupled with geochemical and hydrological measurements and applied to different environments, could shed light on the ecological significance of as yet uncultured microbial taxa.

## Author contributions

CA, BM, and AT designed the experiment. JB and SU collected the samples and carried out the experiments. JB, CA, SU, and AT analyzed the data. JB, CA, SU, BM, and AT wrote the manuscript.

## Funding

This work was made possible through funding from the Eddie and Jo Allison Smith Family Foundation with matching support from UNC's Institute for the Environment and the Wallace Genetic Foundation. CA also received support from a fellowship at the Hanse-Wissenschaftskolleg, Delmenhorst. The funders had no role in designing the study, collecting data, interpreting results, and deciding to publish the work.

### Conflict of interest statement

The authors declare that the research was conducted in the absence of any commercial or financial relationships that could be construed as a potential conflict of interest.
