# Supplementary material for: Riverine Bacterial Communities Reveal Environmental Disturbance Signatures within the Betaproteobacteria and Verrucomicrobia
Source: Front Microbiol. 2016 Sep 15;7:1441. doi: 10.3389/fmicb.2016.01441 (PMC5023673; doi:10.3389/fmicb.2016.01441)
Supplement: Supplementary file 1 [file Table1.DOCX]

**Table S1. Physical, chemical, and biological parameters from the Tar River from (A) Stn. T1 (upstream) and (B) Stn. T6 (downstream)**. The maximum gauge height for Stn. T6 on August 27, 2011 is 2.30 m (source: USGS). DOC, cell counts, and bacterial production data are from a separte study (Bullock 2014).

|  | 1. **Tar River Upstream (T1)** | | | | | |  |
| --- | --- | --- | --- | --- | --- | --- | --- |
|  | | **Nov 2010** | **Feb 2011** | **Jun 2011** | **Sep  2011** | **Nov 2011** | |
| **Precipitation in last 3 days?** | | Yes | No | No | Yes | No | |
| **Max. discharge (m^3^ s^-1^)** | | 0.19 | 0.42 | 0.06 | 0.40 | n/a | |
| **Min. discharge (m^3^ s^-1^)** | | 0.17 | 0.42 | 0.05 | 0.26 | n/a | |
| **Max. gauge height (m)** | | 0.44 | 0.50 | 0.38 | 0.49 | n/a | |
| **Min. gauge height (m)** | | 0.37 | 0.50 | 0.38 | 0.45 | n/a | |
| **Dissolved oxygen (mg L^-1^)** | | n/a | 11.33 | n/a | 5.57 | 7.68 | |
| **Salinity (ppt)** | | n/a | 0.07 | n/a | 0.03 | 0.05 | |
| **pH** | | 7.1 | 7.34 | n/a | 7.12 | 7.56 | |
| **Temperature (°C)** | | 13.9 | 5.39 | n/a | 22.86 | 9.28 | |
| **Bacterial production  (µg C L^-1^ hr^-1^)** | | n/a | 1.03 | 4.75 | 3.73 | 5.86 | |
| **Cell Count (x10^8^ L^-1^)** | | 7.72 | 11.56 | 14.37 | 5.41 | 2.52 | |
| **DOC* (µmol L^-1^)** | | 965 | 451 | 515 | 907 | n/a | |
| **Total Nitrate (µmol L^-1^)** | | 101 | 34.4 | 19.7 | 46.1 | n/a | |

**Table S1. Physical, chemical, and biological parameters from the Tar River from (A) Stn. T1 (upstream) and (B) Stn. T6 (downstream)**. The maximum gauge height for Stn. T6 on August 27, 2011 is 2.30 m (source: USGS). DOC, cell counts, and bacterial production data are from a separte study (Bullock 2014).

|  | 1. **Tar River Downstream (T6)** | | | | |  |
| --- | --- | --- | --- | --- | --- | --- |
|  | **Nov 2010** | **Feb 2011** | **Jun 2011** | **Aug 2011** | **Sep  2011** | **Nov 2011** |
| **Precipitation in last 3 days?** | Yes | No | No | Yes | Yes | No |
| **Max. gauge height (m)** | 0.59 | 0.07 | 0.53 | 0.81 | 0.64 | 0.31 |
| **Min. gauge height (m)** | 0.31 | -0.20 | 0.22 | 0.65 | 0.38 | 0.09 |
| **Dissolved oxygen (mg L^-1^)** | 10.61 | n/a | 7.10 | 2.12 | 2.11 | 8.47 |
| **Salinity (ppt)** | 0.03 | n/a | 1.96 | 0.08 | 0.08 | 0.08 |
| **pH** | 6.71 | n/a | 7.74 | 6.63 | 6.96 | 7.54 |
| **Temperature (°C)** | 13.9 | n/a | 28.73 | 24.69 | 27.03 | 14.02 |
| **Bacterial production  (µg C L^-1^ hr^-1^)** | n/a | 2.15 | 5.31 | n/a | 4.34 | n/a |
| **Cell Count (x10^8^ L^-1^)** | 9.8 | 15.16 | 2.06 | n/a | 1.5 | 3.3 |
| **DOC* (µmol L^-1^)** | 892 | 717 | 730 | n/a | 1710 | n/a |
| **Total Nitrate (µmol L^-1^)** | 33.0 | 33.5 | 15.9 | n/a | 50.1 | n/a |
