# Supplementary material for: Riverine Bacterial Communities Reveal Environmental Disturbance Signatures within the Betaproteobacteria and Verrucomicrobia
Source: Front Microbiol. 2016 Sep 15;7:1441. doi: 10.3389/fmicb.2016.01441 (PMC5023673; doi:10.3389/fmicb.2016.01441)
Supplement: Supplementary file 2 [file Table2.DOCX]

**Table S2. R^2^ and P values of PerMANOVA.** Statistical analyses were done with the Bray-Curtis and Morisita-Horn dissimilarity indices for comparison.

|  |  |  |  |  |  |  |
| --- | --- | --- | --- | --- | --- | --- |

|  | Bray-Curtis | | Morisita-Horn | | |  |
| --- | --- | --- | --- | --- | --- | --- |
|  | R^2^ | *P* | | R^2^ | *P* | |
| Sampling site | 0.17964 | *0.013 | | 0.1815 | **0.007 | |
| Hurricane status | 0.27989 | **0.003 | | 0.38874 | **0.004 | |
| Salinity | 0.18031 | *0.037 | | 0.25711 | *0.026 | |
| Dissolved Oxygen | 0.11227 | 0.229 | | 0.06203 | 0.217 | |
| pH | 0.08417 | 0.421 | | 0.04233 | 0.398 | |
