# Supplementary figures and images for: Riverine Bacterial Communities Reveal Environmental Disturbance Signatures within the Betaproteobacteria and Verrucomicrobia
Source: Front Microbiol. 2016 Sep 15;7:1441. doi: 10.3389/fmicb.2016.01441 (PMC5023673; doi:10.3389/fmicb.2016.01441)

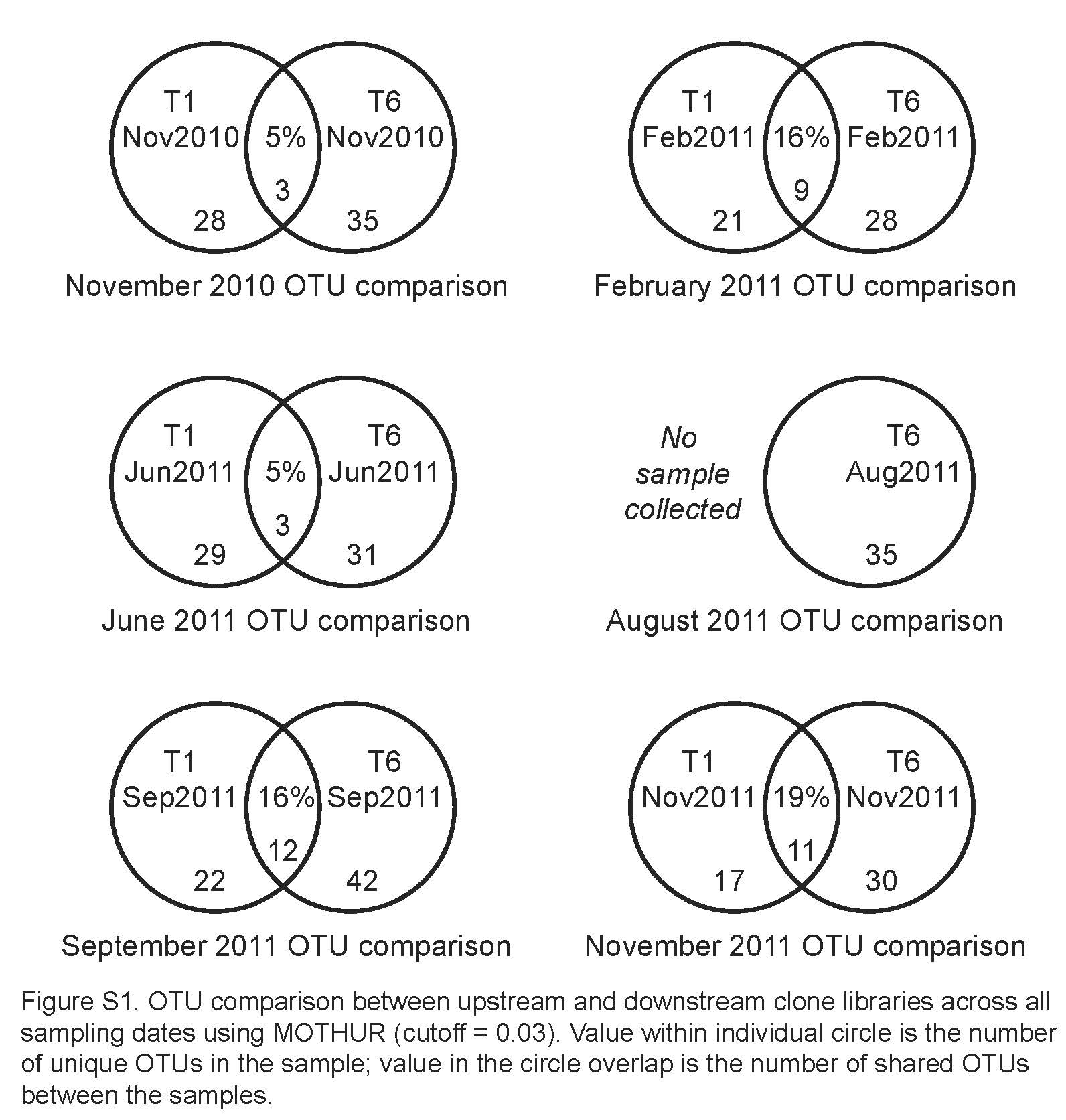

Supplement: Supplementary file 3 [file Image1.JPEG]

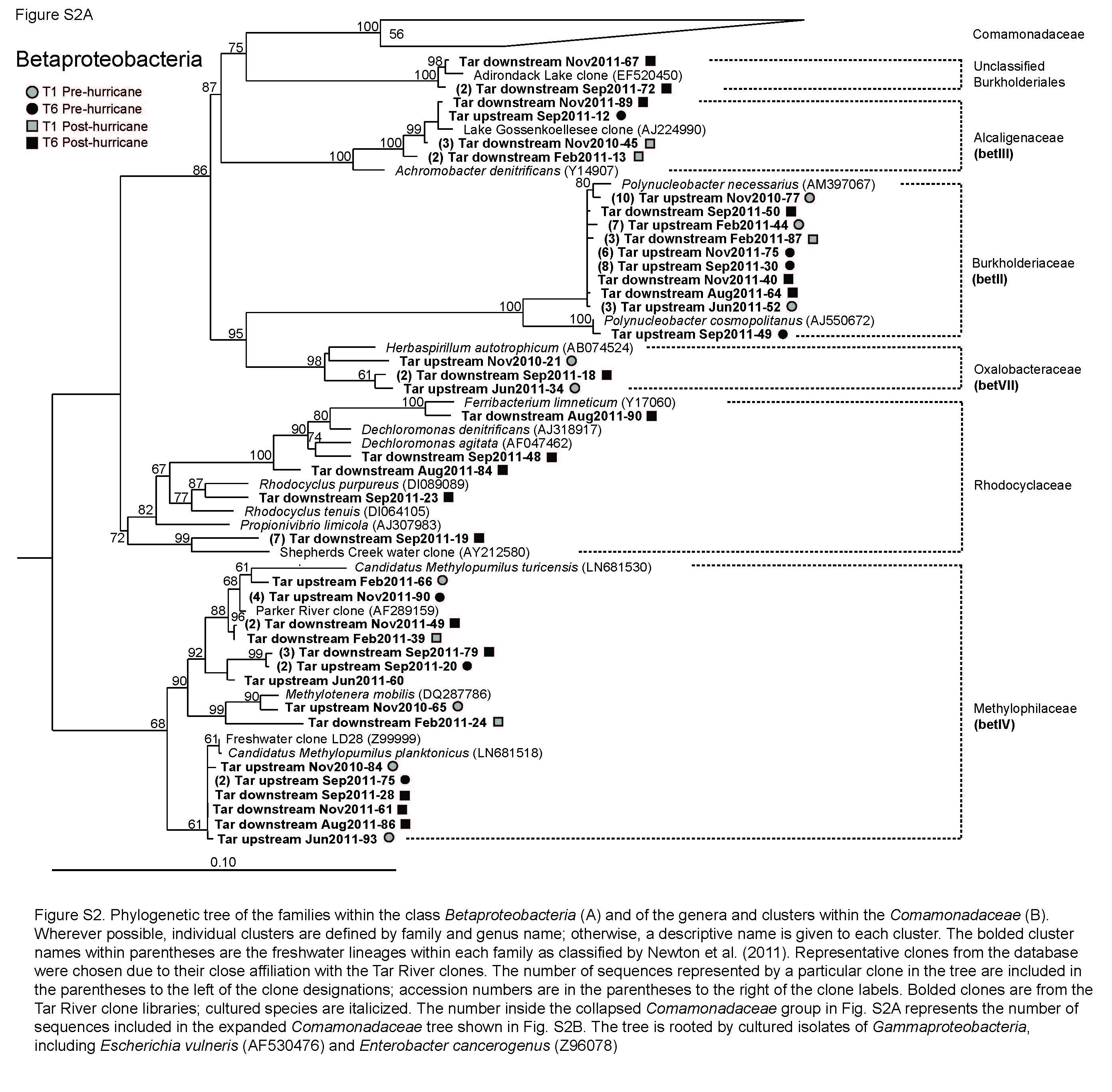

Supplement: Supplementary file 4 [file Image2.JPEG]

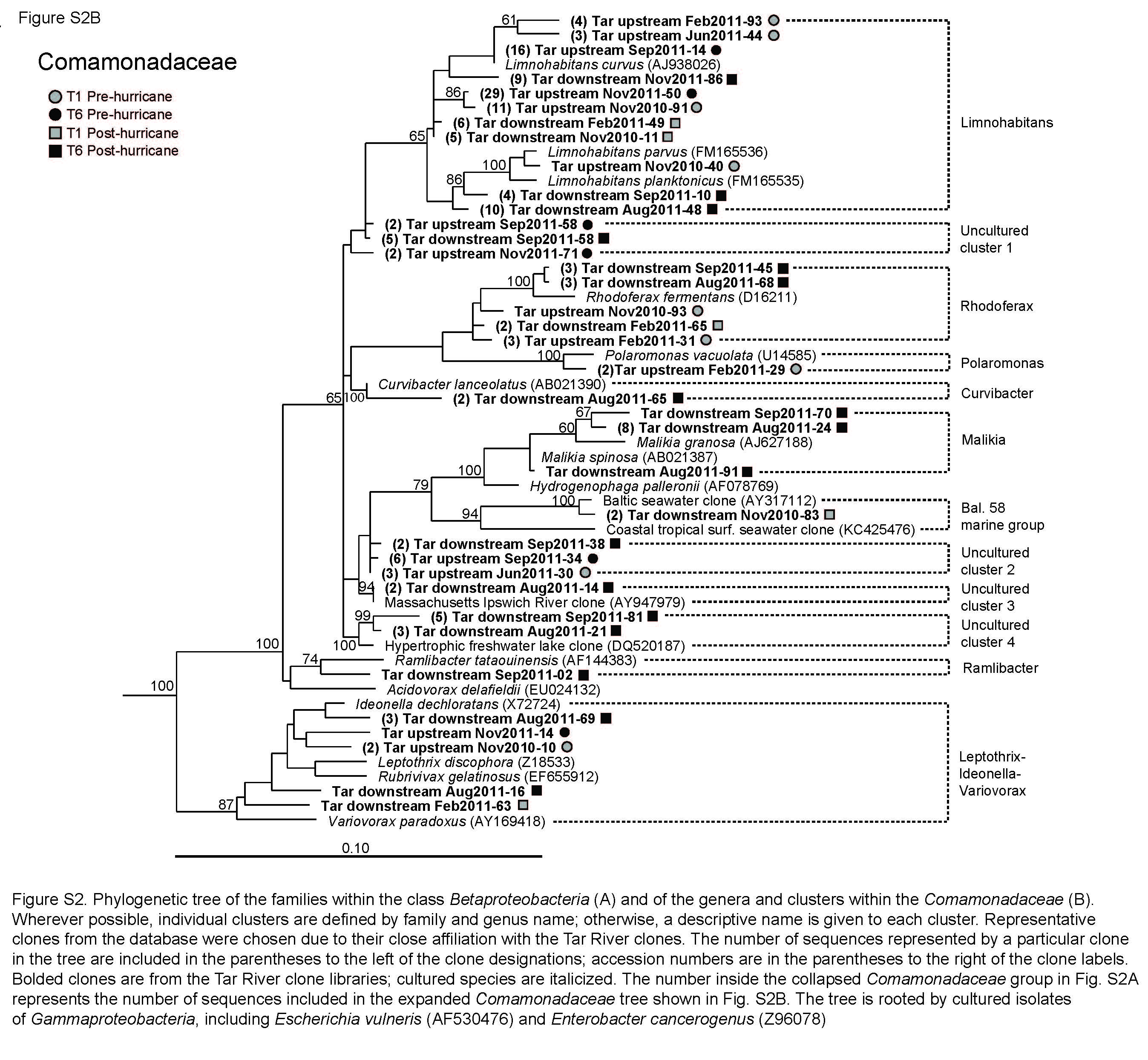

Supplement: Supplementary file 5 [file Image3.JPEG]

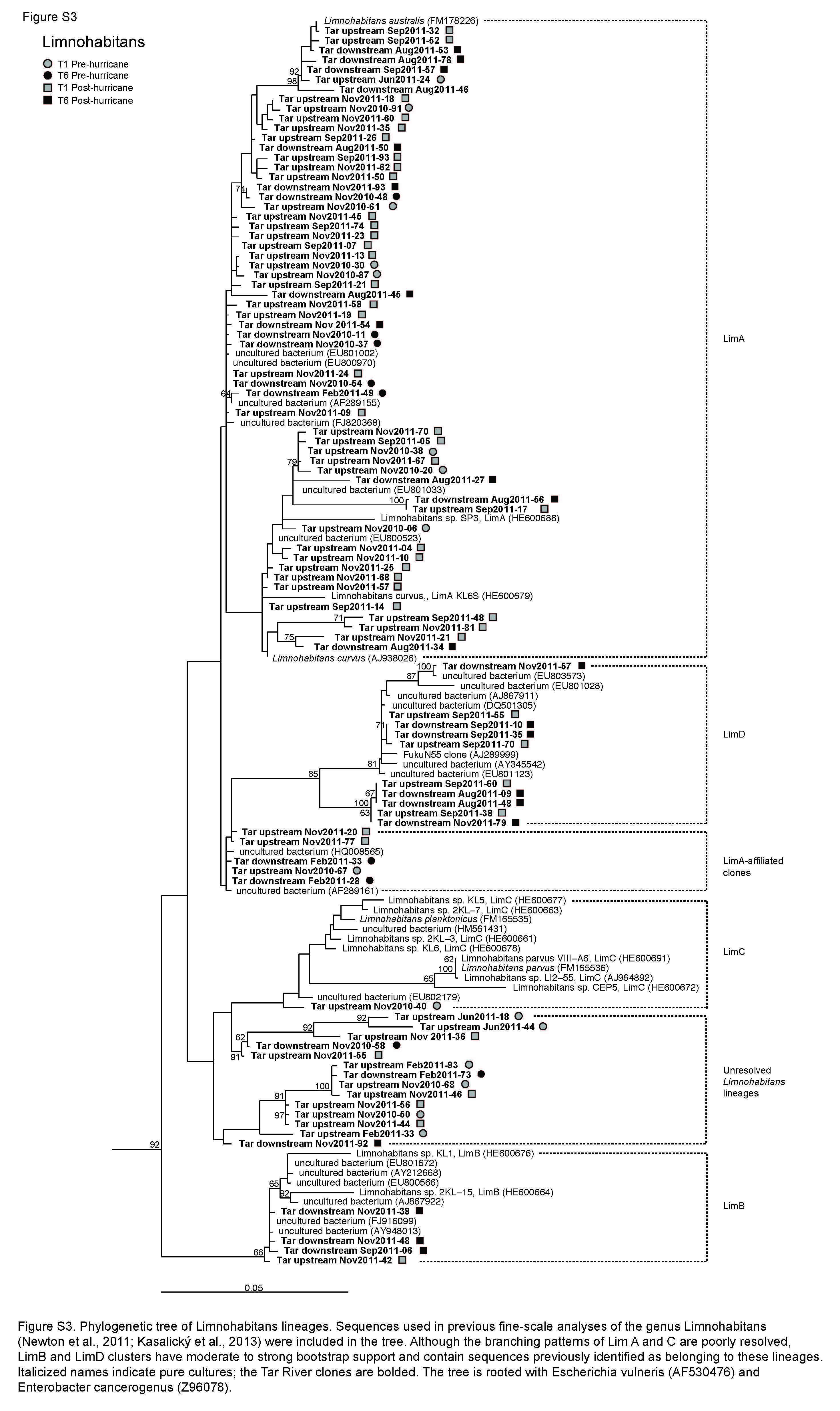

Supplement: Supplementary file 6 [file Image4.JPEG]

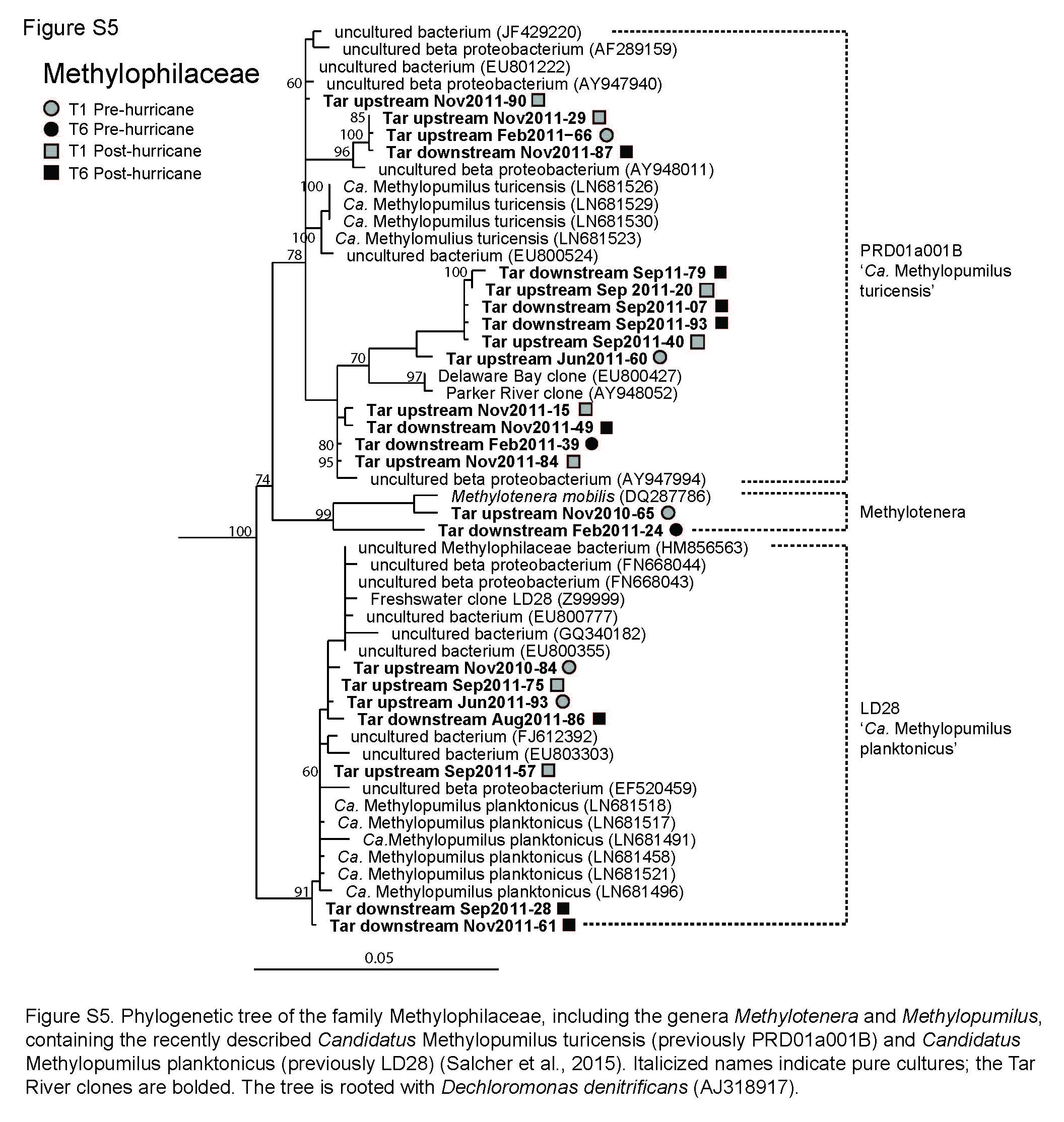

Supplement: Supplementary file 7 [file Image5.jpg]

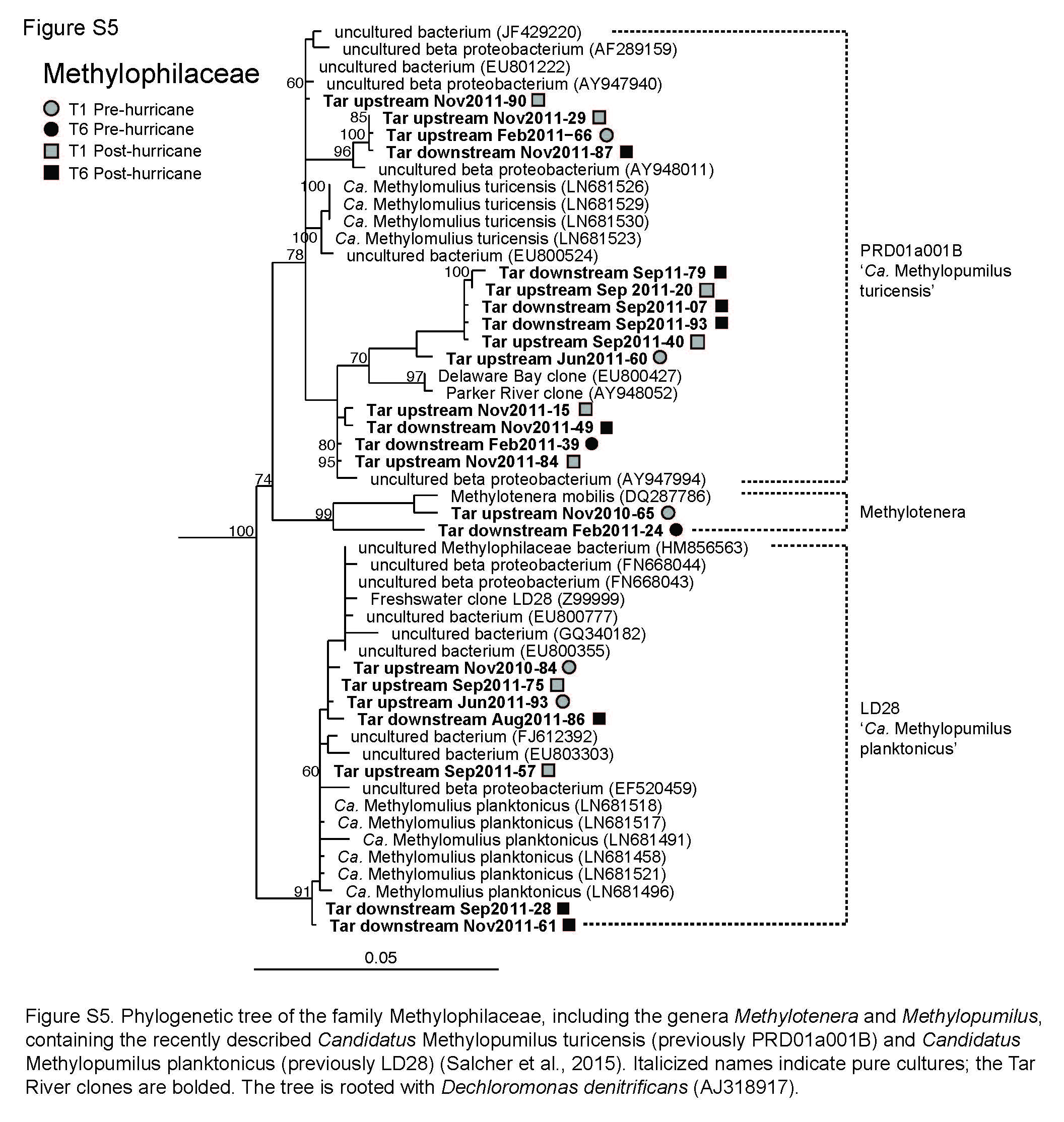

Supplement: Supplementary file 8 [file Image6.JPEG]
